# Supplementary material for: Genetic predictors of participation in optional components of UK Biobank
Source: Nat Commun. 2021 Feb 9;12:886. doi: 10.1038/s41467-021-21073-y (PMC7873270; doi:10.1038/s41467-021-21073-y)
Supplement: Supplementary file 1 — Supplementary Information [file 41467_2021_21073_MOESM1_ESM.pdf]

Genetic predictors of participation in optional components of UK Biobank

Tyrrell et al.

Supplementary figure 1: Manhattan plots of the UK Biobank participation measures from the BOLT-LMM GWAS. A) Food frequency questionnaire, B) Physical Activity, C) Mental health questionnaire and D) aide memoire

A)

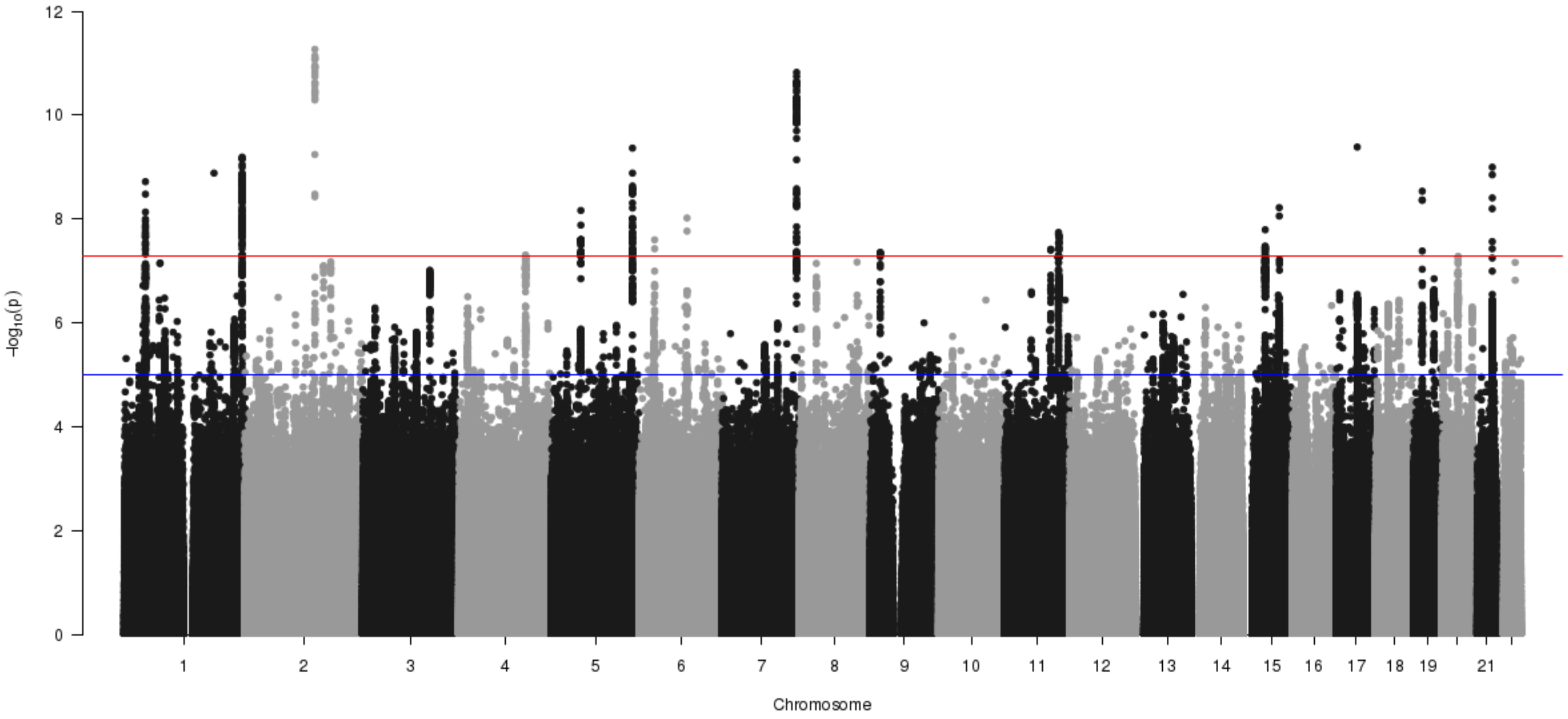

B)

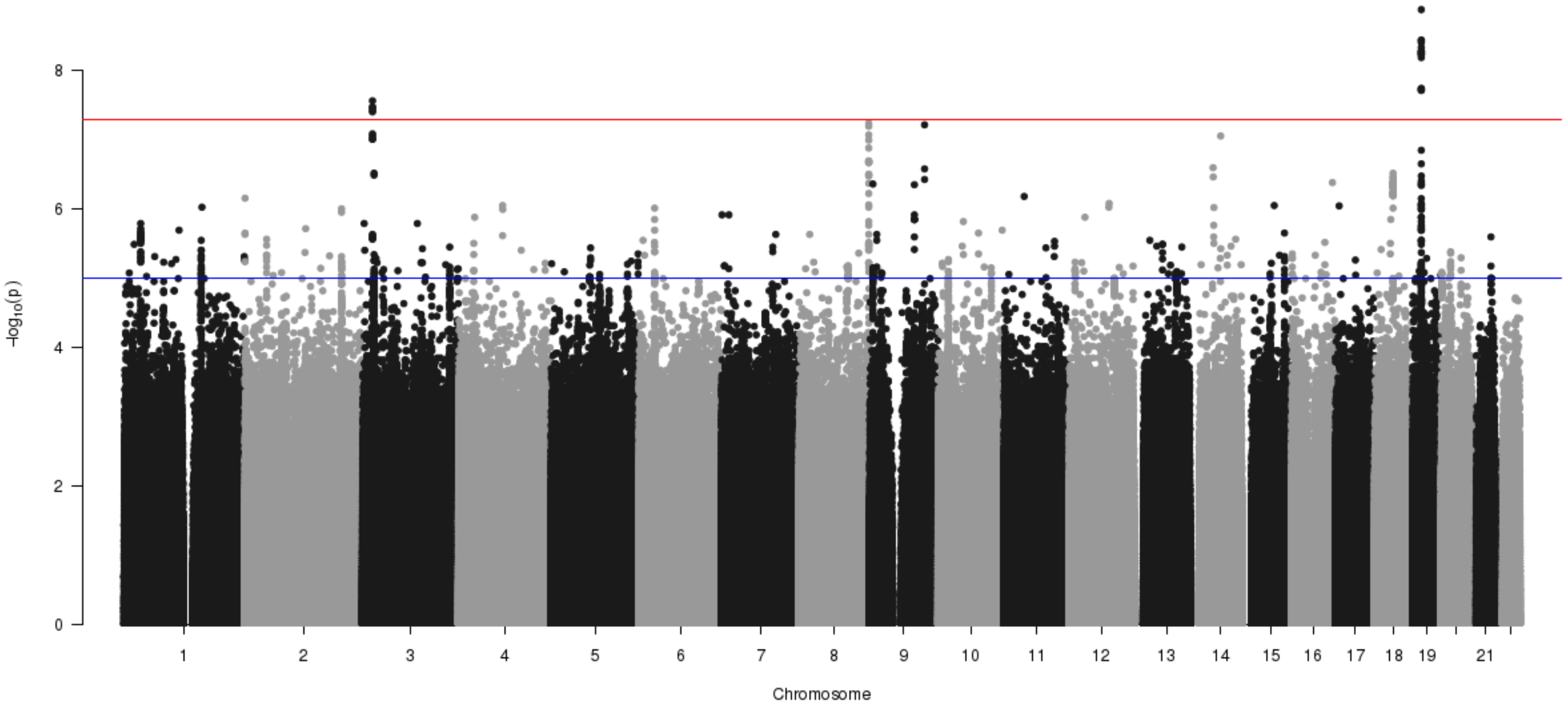

8
6
4
2
0
-log10(p)
1
2
3
4
5
6
7
8
9
10
11
12
13
14
15
16
17
18
19
21
Chromosome

C)

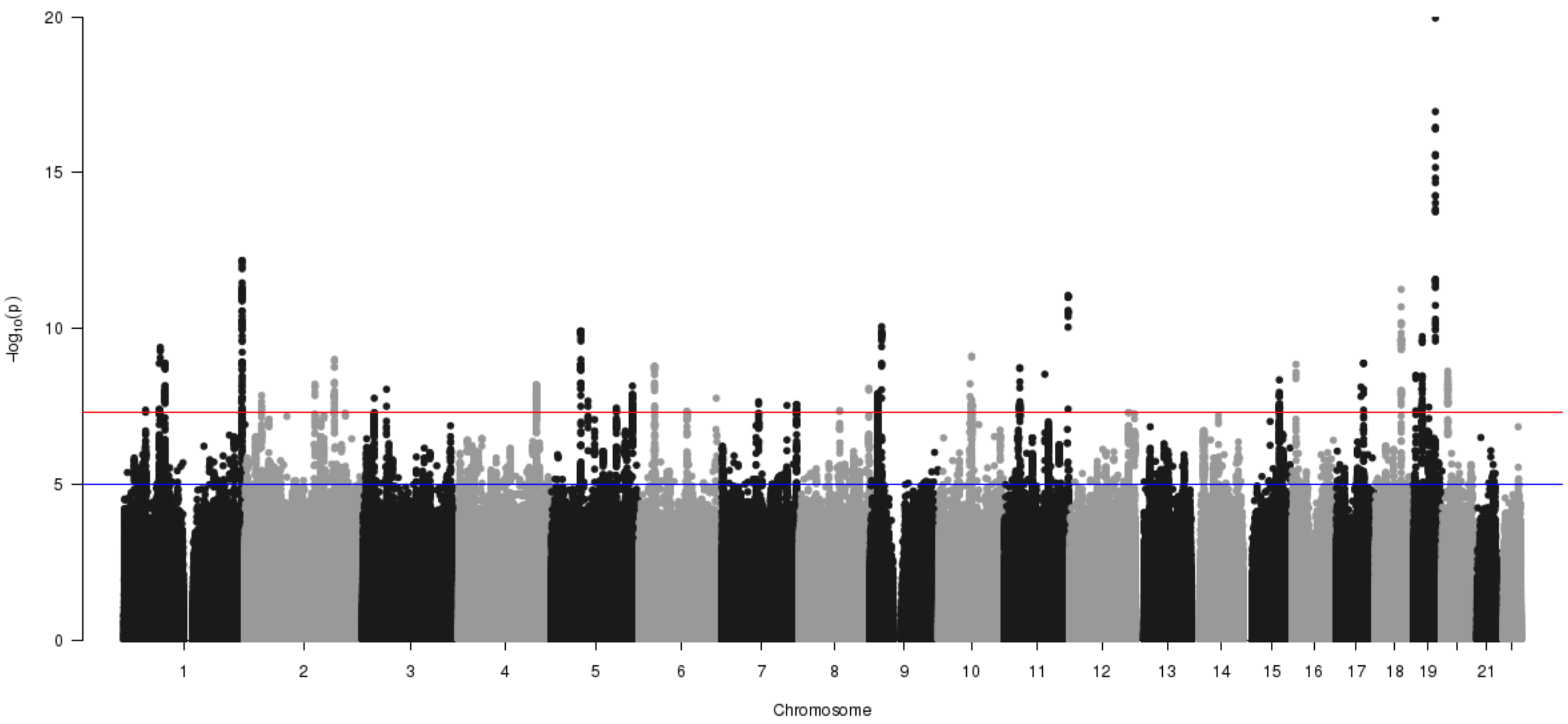

20
15
10
5
0
$-\log_{10}(p)$
1
2
3
4
5
6
7
8
9
10
11
12
13
14
15
16
17
18
19
21
Chromosome

D)

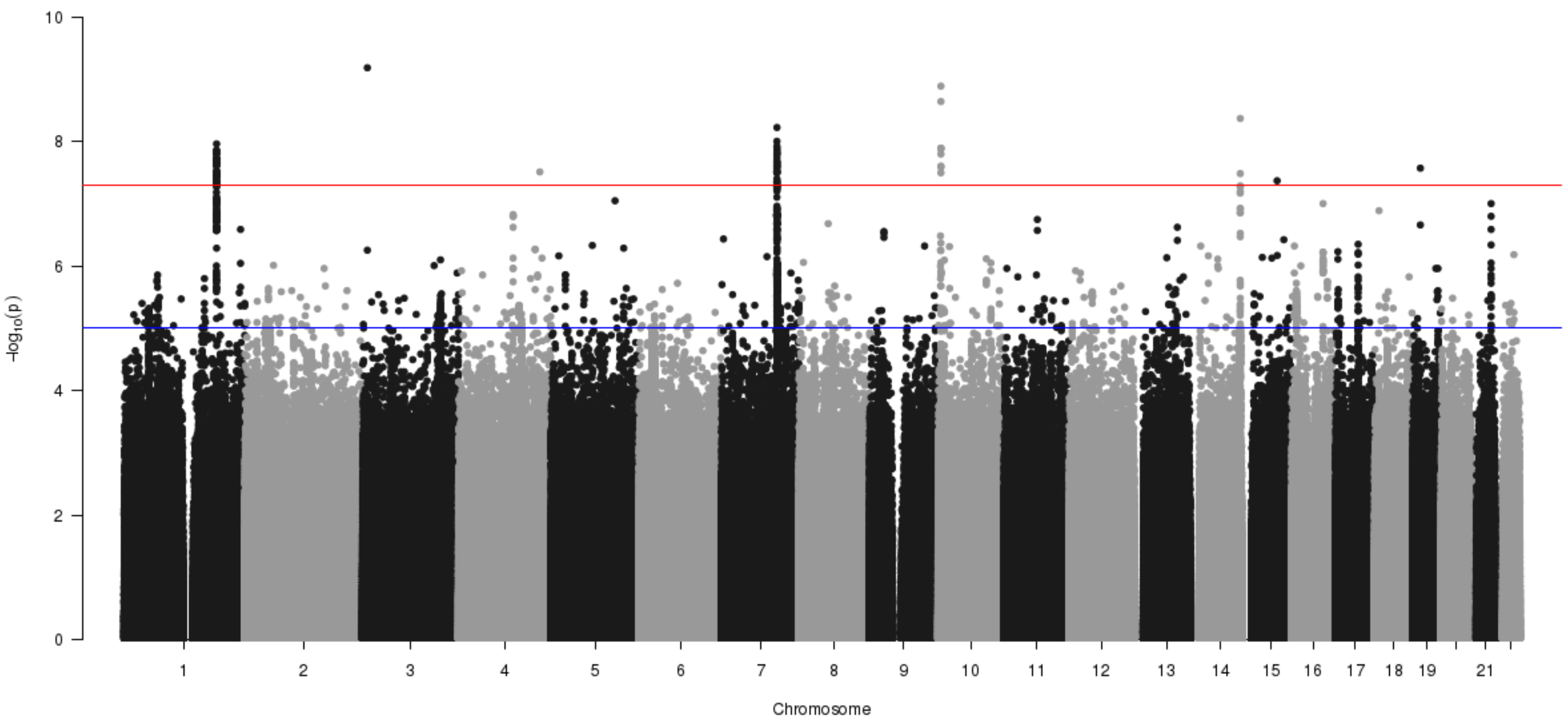

10
8
6
4
2
0
-log10(p)
1
2
3
4
5
6
7
8
9
10
11
12
13
14
15
16
17
18
19
21
Chromosome

Supplementary table 1: Odds ratio (95%CI) for participation in the different components of the UK Biobank if participated in another component

	Participation predictor			
Participation Outcome	FFQ	Physical activity	MHQ	Aide memoire
FFQ	NA	-	-	-
Physical activity	3.73 (3.65, 3.80)	NA	-	-
MHQ	4.45 (4.37, 4.52)	6.66 (6.53, 6.80)	NA	-
Aide memoire	1.21 (1.19, 1.24)	1.17 (1.14, 1.19)	1.20 (1.18, 1.22)	NA

*Results from age and sex adjusted logistic regression. All $P<1x10^{-15}$

Supplementary table 2: Genetic correlations between the different participation measures and whether participants were invited to one of the three optional components in the UK Biobank

Trait1	Trait2	rg	se	z	p	h2_obs	h2_obs_se	h2_int	h2_int_se	gcov_int	gcov_int_se
Aide memoire	MHQ	0.1593	0.0388	4.1049	4.05E-05	0.0549	0.0021	1.0337	0.0095	0.047	0.005
Aide memoire	FFQ	0.3543	0.0417	8.5021	1.86E-17	0.0422	0.0018	1.0338	0.0096	0.0448	0.0051
Aide memoire	Physical activity	0.1603	0.0652	2.4562	0.014	0.0152	0.0012	1.0137	0.0064	0.0308	0.0048
Aide memoire	Invited to participate in MHQ, FFQ and Physical activity	0.0482	0.0445	1.083	0.2788	0.0116	0.0013	1.0112	0.0065	0.0355	0.0055
MHQ	FFQ	0.9172	0.0135	67.7116	0	0.0422	0.0018	1.0338	0.0096	0.4048	0.008
MHQ	Physical activity	0.8469	0.0309	27.4298	1.21E-165	0.0152	0.0012	1.0137	0.0064	0.3267	0.0063
MHQ	Invited to participate in MHQ, FFQ and Physical activity	0.8557	0.0132	64.8374	<1E-250	0.0546	0.0022	1.0363	0.0098	0.4258	0.0076
FFQ	Physical activity	0.8713	0.0313	27.8195	2.52E-170	0.0152	0.0012	1.0137	0.0064	0.2888	0.0056
FFQ	Invited to participate in MHQ, FFQ and Physical activity	0.651	0.0217	30.0488	2.26E-198	0.0416	0.0019	1.0387	0.0101	0.0137	0.0059
Physical activity	Invited to participate in MHQ, FFQ and Physical activity	0.6102	0.0375	16.264	1.78E-59	0.0152	0.0012	1.0139	0.0065	0.0057	0.0057

rg - genetic correlation; se - standard error of genetic correlation; z - z-score of the genetic correlation; p - p-value of genetic correlation (two-sided and no adjustment for multiple comparisons, but only those reaching Bonferroni significance are reported);
h2_obs - observed SNP heritabiliity of trait 2; h2_obs_se - standard error of SNP heritability; h2_int - the intercept term of the univariate LDSC of trait 2; h2_int_se - standard error of intercept term
gcov_int - the pehnotypic correlation between trait 1 and trait 2 after accounting for sample overlap; gcov_int_se - standard error of gcov_int

Supplementary table 3: Demographics of those invited to participate in at least one optional survey versus those not invited to participate

Demographic	Invited to at least one optional survey	Not invited to any optional surveys	Odds (95%CI) of receiving an invitation to participate in optional surveys per unit change in demographic*	P**
N	336,633	114,464		
Mean age at baseline (SD)	56.7 (8.0)	59.1 (7.9)	0.96 (0.96, 0.96)	$<1x10^{-15}$
Male sex, N (%)	155,174 (46.1)	51,077 (44.6)	1.07 (1.06, 1.09)	$<1x10^{-15}$
Mean BMI at baseline (SD)	27.2 (4.7)	28.0 (5.0)	0.84 (0.83, 0.85)	$<1x10^{-15}$
Mean Townsend deprivation index (SD)	-1.71 (2.8)	-0.77 (3.3)	0.50 (0.48, 0.51)	$<1x10^{-15}$
Mean years in education (SD)***	15.6 (4.8)	12.5 (5.3)	1.10 (1.09, 1.10)	$<1x10^{-15}$
Mean systolic blood pressure (SD)	143 (24)	149 (25)	0.85 (0.84, 0.86)	$<1x10^{-15}$
Mean fluid intelligence (SD)	6.0 (2.1)	4.8 (1.9)	1.48 (1.47, 1.49)	$<1x10^{-15}$
Mean self reported physical activity	7.4 (1.1)	7.4 (1.2)	0.91 (0.90, 0.92)	$<1x10^{-15}$
Mean physical activity (accelerometer)****	0.21 (0.07)	NA	NA	NA
Smoking status, N (%)				
Never	185,195 (55.0)	57,512 (50.2)	Reference	Reference
Former	121,357 (36.1)	38,561 (33.7)	1.06 (1.04, 1.07)	$1x10^{-13}$
Current	26,015 (7.7)	16,367 (14.3)	0.45 (0.44, 0.46)	$<1x10^{-15}$
Type 2 diabetes, N (%)	14,378 (4.3)	8,185 (7.2)	0.68 (0.66, 0.70)	$<1x10^{-15}$
Depression, N (%)	40,234 (12.0)	8,850 (7.7)	1.32 (1.30, 1.33)	$<1x10^{-15}$

*For all continuous measures this is per SD change except for age and educational years which are per year

**P-value from logistic regression, adjusted for age, sex and assessment centre

Supplementary table 8: List of predictor traits from 8 different categories used in Mendelian randomization.

Test trait	Category	N SNPs in 2-sample MR	Primary GWAS reference	Notes
BMI	Anthropometric	72	Locke et al., 2015	
Body fat percentage	Anthropometric	10	Lu et al., 2016	
Bone mineral denisty (femur neck)	Anthropometric	50	Estrada et al., 2012	
Bone mineral denisty (lumbar spine)	Anthropometric	48	Estrada et al., 2012	
Favourable adiposity	Anthropometric	14	Ji et al., 2019	
Forced vital capacity	Anthropometric	6	Loth et al., 2014	
Height	Anthropometric	809	Wood et al., 2014	
Maternal birthweight	Anthropometric	9	Beaumont et al., 2018	
Waist hip ratio	Anthropometric	315	Pulit et al., 2018	
Waist hip ratio (adjusted for BMI)	Anthropometric	345	Pulit et al., 2018	
Age lost virginity	Behavioural	29	Day et al., 2016	
Alcohol consumption	Behavioural	8	Clarke et al., 2017	UK Biobank used in discovery
Caffeine	Behavioural	6	Coffee and Caffeine Genetics Consortium, 2015	
Chronotype	Behavioural	46	Jones et al., 2019	
Extraversion	Behavioural	4	Lo et al., 2017	
Risk taking	Behavioural	13	UK Biobank analyses in 120,000	
Smoking	Behavioural	4	The Tobacco and Genetics Consortium, 2010	

Alkaline phosphatase (ALP)	Biomarkers, metabolites and cellular features	14	Chambers et al., 2011
C-reactive protein (CRP)	Biomarkers, metabolites and cellular features	14	Dehghan et al., 2011
Calcium	Biomarkers, metabolites and cellular features	7	O'Seaghdha et al., 2013
Estimated glomerula filtration rate (GFR)	Biomarkers, metabolites and cellular features	50	Pattaro et al., 2015
Ferritin	Biomarkers, metabolites and cellular features	6	Benyamin et al., 2014
Gamma glutamyl transferase (GGT)	Biomarkers, metabolites and cellular features	25	Chambers et al., 2011
Haemoglobin	Biomarkers, metabolites and cellular features	11	Danjou et al., 2015
Iron levels	Biomarkers, metabolites and cellular features	5	Benyamin et al., 2014
Mean cell haemoglobin	Biomarkers, metabolites and cellular features	27	Astle et al., 2016
Mean cell volume	Biomarkers, metabolites and cellular features	23	Astle et al., 2016
Red blood cell count	Biomarkers, metabolites and cellular features	10	Astle et al., 2016
Telomere length	Biomarkers, metabolites and cellular features	7	Codd et al., 2013
Thyroid stimulating hormone (TSH)	Biomarkers, metabolites and cellular features	20	Porcu et al., 2013
Transferrin	Biomarkers, metabolites and cellular features	9	Benyamin et al., 2014
Transferrin saturation	Biomarkers, metabolites and cellular features	5	Benyamin et al., 2014

Urate	Biomarkers, metabolites and cellular features	19	Kottgen et al., 2012	
Vitamin D	Biomarkers, metabolites and cellular features	6	Jiang, et al, 2018	
Breast cancer	Cancer	75	Michailidou et al., 2015	Women only
Colorectal cancer	Cancer	36	Schumacher et al., 2015	
Prostate cancer	Cancer	57	Almi Al Olama et al., 2015	Men only
2 hour glucose	Cardiovascular and metabolic markers	8	Scott et al., 2012	
Coronary artery disease (CAD)	Cardiovascular and metabolic markers	64	Nikpay et al., 2015	
Diastolic blood pressure (DBP)	Cardiovascular and metabolic markers	109	Ehret et al., 2016; International Consortium for Blood Pressure GWAS, 2011; Warren et al., 2017; Surendran et al., 2016	
Fasting glucose	Cardiovascular and metabolic markers	33	Scott et al., 2012	
Fasting insulin	Cardiovascular and metabolic markers	14	Scott et al., 2012	
Fasting insulin adjusted for BMI	Cardiovascular and metabolic markers	12	Scott et al., 2012	
HDL cholesterol	Cardiovascular and metabolic markers	98	Global Lipids Genetics Consortium (http://lipidgenetics.org)	
Homocysteine	Cardiovascular and metabolic markers	13	van Meurs et al., 2013	
IgA nephropathy	Cardiovascular and metabolic markers	15	Kiryluk et al., 2014	
Insulin resistance	Cardiovascular and metabolic markers	53	Lotta et al., 2017	

Insulin secretion	Cardiovascular and metabolic markers	11	Dimas et al., 2014	
LDL cholesterol	Cardiovascular and metabolic markers	80	Global Lipids Genetics Consortium (http://lipidgenetics.org)	
Leptin adjusted for BMI	Cardiovascular and metabolic markers	9	Kilpelainen et al., 2016	
Pulse pressure	Cardiovascular and metabolic markers	44	Ehret et al., 2016; International Consortium for Blood Pressure GWAS, 2011; Warren et al., 2017; Surendran et al., 2016	
Resting heart rate	Cardiovascular and metabolic markers	71	Eppinga et al., 2016	
Stroke	Cardiovascular and metabolic markers	6	Malik et al., 2018	
Systolic blood pressure (SBP)	Cardiovascular and metabolic markers	89	Ehret et al., 2016; International Consortium for Blood Pressure GWAS, 2011; Warren et al., 2017; Surendran et al., 2016	
Triglycerides	Cardiovascular and metabolic markers	62	Global Lipids Genetics Consortium (http://lipidgenetics.org)	
Type 2 diabetes	Cardiovascular and metabolic markers	89	Mahajan et al., 2018	
Educational attainment	Education and cognition	69	Okbay et al., 2016	SNPs were those at GWS excluding UK Biobank
intelligence	Education and cognition	14	Sniekers et al., 2017	
Age related macular degeneration	Other diseases	28	Fritsche et al., 2016	
Crohn's disease	Other diseases	142	Liu et al., 2015	

Irritable bowel disease	Other diseases	158	Liu et al., 2015	
Migraine	Other diseases	33	Gormley et al., 2016	
Motion sick	Other diseases	35	Hromatka et al., 2015	
Osteoarthritis	Other diseases	5	Evangelou et al., 2012	
Restless legs	Other diseases	19	Schormair et al., 2017	
Ulcerative colitis	Other diseases	87	Liu et al., 2015	
Alzheimer's disease	Psychological and neurological conditions	21	Lambert et al., 2013	
Bipolar disorder	Psychological and neurological conditions	21	https://www.biorxiv.org/content/10.1101/173062v4	Data from BioRXiv and PGC consortium
Depression	Psychological and neurological conditions	17 and 38	Hyde et al., 2016 and Wray et al., 2018	Wray et al., discovery included UK Biobank
Neuroticism	Psychological and neurological conditions	9	Genetics of Personality Consortium et al., 2015	
Schizophrenia	Psychological and neurological conditions	137	Pardinas et al., 2018	
DHEAS	Reproductive traits	8	Zhai et al., 2011	
Endometriosis	Reproductive traits	14	Sapkota et al., 2017	Women only
Gestational duration	Reproductive traits	5	Zhang et al., 2017	
Menarche	Reproductive traits	128	Perry et al., 2014	Women only
Menopause	Reproductive traits	56	Day et al., 2015	Women only
Polycystic ovaries (PCOS)	Reproductive traits	6	Day et al., 2015	Women only
Sex hormone binding globulin (SHBG)	Reproductive traits	12	Coviello et al., 2012	
