# Supplementary material for: Genetic predictors of participation in optional components of UK Biobank
Source: Nat Commun. 2021 Feb 9;12:886. doi: 10.1038/s41467-021-21073-y (PMC7873270; doi:10.1038/s41467-021-21073-y)
Supplement: Supplementary file 3 — Descriptions of Additional Supplementary Files [file 41467_2021_21073_MOESM3_ESM.pdf]

Descriptions of Additional Supplementary Files

Supplementary Data 1

Description: Variants identified at genome wide significance for not receiving an invite to participate in at least one of optional components of the UK Biobank

Supplementary Data 2:

Description: Variants associated with the four participation measures from genome wide association analyses in the UK Biobank with 6x10-9 < P < 5x10-8

Supplementary Data 3

Description: Mendelian randomization results for a range of different test traits against the four participation measures.

Supplementary Data 4

Description: Results from 1-sample MR analyses, representing odds of participation per SD higher genetically instrumented cigarettes per day
