# Supplementary material for: Genetic predictors of participation in optional components of UK Biobank
Source: Nat Commun. 2021 Feb 9;12:886. doi: 10.1038/s41467-021-21073-y (PMC7873270; doi:10.1038/s41467-021-21073-y)
Supplement: Supplementary file 8 — Reporting Summary [file 41467_2021_21073_MOESM8_ESM.pdf]

nature research

Corresponding author(s): Jess Tyrrell

Last updated by author(s): Jul 14, 2020



# Reporting Summary

Nature Research wishes to improve the reproducibility of the work that we publish. This form provides structure for consistency and transparency in reporting. For further information on Nature Research policies, see our Editorial Policies and the Editorial Policy Checklist.

## Statistics

For all statistical analyses, confirm that the following items are present in the figure legend, table legend, main text, or Methods section.

| n/a | Confirmed | |
|---|---|---|
| ☐ | ☒ | The exact sample size ($n$) for each experimental group/condition, given as a discrete number and unit of measurement |
| ☐ | ☒ | A statement on whether measurements were taken from distinct samples or whether the same sample was measured repeatedly |
| ☐ | ☒ | The statistical test(s) used AND whether they are one- or two-sided<br>*Only common tests should be described solely by name; describe more complex techniques in the Methods section.* |
| ☐ | ☒ | A description of all covariates tested |
| ☐ | ☒ | A description of any assumptions or corrections, such as tests of normality and adjustment for multiple comparisons |
| ☐ | ☒ | A full description of the statistical parameters including central tendency (e.g. means) or other basic estimates (e.g. regression coefficient) AND variation (e.g. standard deviation) or associated estimates of uncertainty (e.g. confidence intervals) |
| ☐ | ☒ | For null hypothesis testing, the test statistic (e.g. $F$, $t$, $r$) with confidence intervals, effect sizes, degrees of freedom and $P$ value noted<br>*Give P values as exact values whenever suitable.* |
| ☒ | ☐ | For Bayesian analysis, information on the choice of priors and Markov chain Monte Carlo settings |
| ☒ | ☐ | For hierarchical and complex designs, identification of the appropriate level for tests and full reporting of outcomes |
| ☒ | ☐ | Estimates of effect sizes (e.g. Cohen's $d$, Pearson's $r$), indicating how they were calculated |

*Our web collection on statistics for biologists contains articles on many of the points above.*

## Software and code

Policy information about availability of computer code

| | |
|---|---|
| Data collection | No software - data collection was done centrally by UK Biobank |
| Data analysis | R version 3.5.1 and Stata version 16 were used for data analysis. BOLT-LMM was utilised for running the GWAS. |

For manuscripts utilizing custom algorithms or software that are central to the research but not yet described in published literature, software must be made available to editors and reviewers. We strongly encourage code deposition in a community repository (e.g. GitHub). See the Nature Research guidelines for submitting code & software for further information.

## Data

Policy information about availability of data

All manuscripts must include a data availability statement. This statement should provide the following information, where applicable:

- Accession codes, unique identifiers, or web links for publicly available datasets
- A list of figures that have associated raw data
- A description of any restrictions on data availability

All summary GWAS data are available from the authors on request.



# Field-specific reporting

# Life sciences study design

All studies must disclose on these points even when the disclosure is negative.

| | |
|---|---|
| Sample size | Sample size was based on participants in the UK Biobank of European ancestry with genetic data and all questionnaire data available. |
| Data exclusions | Non-European ancestry participants in UKB |
| Replication | No formal replication of the GWAS, although we demonstrated that our measures of participation were strongly correlated with those from another study (ALSPAC) |
| Randomization | Genetic analyses so for Mendelian randomisation participants were randomised based on genotype |
| Blinding | NA to this study design. |

# Reporting for specific materials, systems and methods

We require information from authors about some types of materials, experimental systems and methods used in many studies. Here, indicate whether each material, system or method listed is relevant to your study. If you are not sure if a list item applies to your research, read the appropriate section before selecting a response.

## Materials & experimental systems

| n/a | Involved in the study | |
|---|---|---|
| ☒ | ☐ | Antibodies |
| ☒ | ☐ | Eukaryotic cell lines |
| ☒ | ☐ | Palaeontology and archaeology |
| ☒ | ☐ | Animals and other organisms |
| ☐ | ☐ | Human research participants |
| ☒ | ☐ | Clinical data |
| ☒ | ☐ | Dual use research of concern |

## Methods

| n/a | Involved in the study | |
|---|---|---|
| ☒ | ☐ | ChIP-seq |
| ☒ | ☐ | Flow cytometry |
| ☒ | ☐ | MRI-based neuroimaging |

# Human research participants

Policy information about studies involving human research participants

| | |
|---|---|
| Population characteristics | UK Biobank recruited 500,000 people aged between 40 and 70 who agreed to have their health followed. Detailed information on this cohort is available elsewhere: https://bmjopen.bmj.com/content/6/3/e009161 |
| Recruitment | The UK Biobank participants were recruited between 2006 and 2010 from across the UK. Previous studies have demonstrated the volunteer bias within the UK Biobank and our study focuses on further biases within the optional components of the UKB. |
| Ethics oversight | Ethical approval: UK Biobank has received ethics approval from the National Health Service National Research Ethics Service (ref 11/NW/0382). |

Note that full information on the approval of the study protocol must also be provided in the manuscript.
